# Supplementary material for: A European International Multicentre Survey on the Current Practice of Perioperative Antibiotic Prophylaxis for Paediatric Liver Transplantations
Source: Antibiotics (Basel). 2023 Feb 1;12(2):292. doi: 10.3390/antibiotics12020292 (PMC9952614; doi:10.3390/antibiotics12020292)
Supplement: Supplementary file 1 [file antibiotics-12-00292-s001.zip › antibiotics-2144688-supplementary.pdf]

Supplementary Table S1. Duration and type of antibiotic prophylaxis according to the number of annual transplantations.

		Annual transplantations	
		≤ 20	> 20
No. of centres		11 (55 %)	9 (45 %)
Standard duration of treatment	24-48 hours	3 (27 %)	3 (33 %)
	3-5 days	3 (27 %)	3 (33 %)
	6-10 days	1 (9 %)	2 (22 %)
	Single shot	1 (9 %)	0 (0 %)
	No standard	3 (27 %)	1 (11 %)
Type of standard prophylaxis	Narrow spectrum	7 (64 %)	3 (33 %)
	Broad spectrum	4 (36 %)	6 (66 %)
Availability of infectious disease specialist		10 (91 %)	6 (66 %)

Supplementary Table S2. Infection surveillance strategies and non-antibiotic anti-infective measures.

Surveillance strategies	n (%)
Ascites	11 (61%)
Blood	9 (50%)
Urine	9 (50%)
Tracheal secrete	11 (61%)
Skin swabs	4 (22%)
Rectal swabs	13 (72%)
Nose swabs	2 (11%)
Throat swabs	7 (39%)
Wound swabs	1 (6%)
Preservation fluid of the graft	10 (56%)
All drain fluids	1 (6%)
**Biomarker**	
CRP	19 (95%)
Procalcitonin	13 (65%)
Leukocyte count	12 (60%)
Interleukin 6	2 (10%)
CRP	19 (95%)
**Anti-infective measures**	
**Isolation**	
1 week	1 (5%)
2-3 weeks	1 (5%)
Until discharge	2 (10%)

Until discontinuation and normalisation of CRP and Procalcitonin	1 (5%)
No	14 (70%)
**Other prophylactic measures**	
Chlorhexidine dressing	8 (40%)
Octenidine washing	4 (20%)
Selective bowel decontamination	1 (5%)
Probiotics	1 (5%)
Silver nitrate or antibiotic-impregnated central catheter	1 (5%)
No standard	2 (10%)
**Antifungal prophylaxis in the absence of risk factors**	12 (60%)
**Agent**	
Fluconazole	4 (20%)
Liposomal amphotericin b	1 (5%)
Micafungin	2 (10%)
Caspofungin	1 (5%)
Total	20 (100%)
**Duration of prophylaxis**	
3-7 days	12 (60%)
14-28 days	2 (10%)
> 28 days	1 (5%)
No fungal prophylaxis	5 (25%)
Total	20 (100%)
**Additional risk factors triggering antifungal prophylaxis**	
Antibiotic pre-treatment	2 (14%)
Pre-existing conditions	5 (33%)
Positive fungal cultures	12 (80%)
Intraabdominal patch	5 (33%)
Reoperations	7 (47%)
Massive blood transfusion	3 (20%)
Course of CrP	5 (33%)
Course of procalcitonin	3 (20%)
Acute liver failure	1 (7%)
Length of hospital stay	1 (7%)
Total	15 (100%)
**Antiviral prophylaxis**	
**Agent**	
Gancicovir	7 (35%)
Aciclovir	7 (35%)
Aciclovir or Ganciclovir	1 (5%)
Unspecific immunoglobulins	1 (5%)
Pre-emptive therapy in case of viraemia or symptoms	3 (15%)
Total	20 (100%)
**Risk constellation**	
All patients	7 (41%)
CMV+donor	4 (24%)
CMV+recipient	3 (18%)
Mismatch (CMV-recipient)	2 (12%)
Recipient age under one year	1 (6%)
Total	17 (100%)

CrP = c-reactive protein; CMV+/- = cytomegaly virus positive/negative

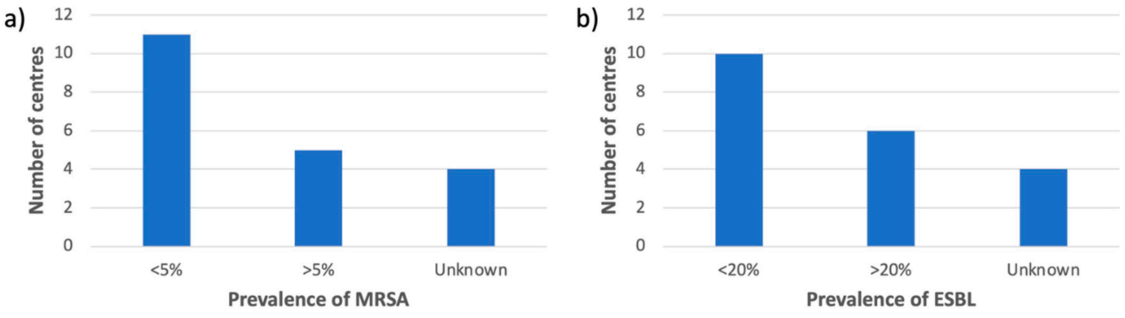

Supplementary Figure S1. Prevalence of multidrug resistant bacteria in participating paediatric liver transplantation centres. (a) Methicillin-resistant *staphylococcus aureus*. (b) Extended-spectrum ß-lactamase.

Questionnaire:

1. Please mark your region in Europe.

- Central Europe
- Northern Europe
- Southern Europe
- Western Europe
- Eastern Europe

2. Please indicate the average number of paediatric liver transplantation per year at your hospital.

- <5
- 5-10
- 11-20
- 21-30
- >30

3. Please indicate the average number of high urgency transplantations

- <3
- 3-5
- >5

4. Please indicate the average number of living related liver transplantations per year

- <5
- 5-10
- 11-15
- 16-20

- >20

5. Is the prevalence of MRSA known? Can you specify please?

- <5%
- 5-10%
- 10-20%
- 20-30%
- >30%
- unknown

6. Is the prevalence of ESBL/Extended spectrum ß-lactamases known? Can you please specify?

- <5%
- 5-10%
- 10-20%
- 20-30%
- >30%
- unknown

7. Are you using any of the mentioned anti-infective or antiseptic measures routinely in pediatric liver transplant patients (several answers can be chosen)?

- Chlorhexidine dressing
- Octenidine washing
- Selective bowel decontamination
- Silver nitrate or antibiotic impregnated central catheters
- Probiotics
- other: ______________________

8. Infectious management/work-up of perioperative anti-infective measures is determined by…(several answers can be chosen)

- Surgeon
- Paediatric surgeon
- Gastroenterologist
- Paediatric gastroenterologist
- Intensive care specialist
- Paediatric intensive care specialist
- Anaesthetist
- Paediatric anaesthetist
- Infectious disease specialist
- Paediatric infectious disease specialist
- other: ______________________

9. Which unit is in charge of the postoperative care? (several answers can be chosen)

- Paediatric intensive care unit
- Adult intensive care unit
- Mixed intensive care unit
- other: ______________________

10. Do you provide a basiliximab based immunosuppression?

- Yes
- No

11. Which is your standard immunosuppression regimen in the first three weeks?

- Steroids plus tacrolimus plus mycophenolic mofetil
- Steroids plus cyclosporine plus mycophenolic mofetil
- Steroids plus tacrolimus
- Steroids plus cyclosporine
- Steroids plus mycophenolic mofetil
- Tacrolimus plus mycophenolic mofetil
- Cyclosporine plus mycophenolic mofetil
- Steroids alone
- Tacrolimus alone
- Cyclosporine alone
- Other regimen: ______________________

12. The target trough level of Tacrolimus in the first week is:

- <8 µg/l
- 8-12 µg/l
- 12-16 µg/l
- >16 µg/l

13. The target of the Cyclosporin A trough level in the first week is:

- <225 µg/l
- 225-300 µg/l
- >300 µg/l

14. Which is the preferred initial steroid?

- Prednisolone
- Methylprednisolone
- Dexamethasone
- Other: ______________________

15. What is the initial daily steroid dosage?

- ______________________ mg
- ______________________ mg/kg
- ______________________ mg/m2

16. Which antibiotic regimen is used as perioperative standard?

- Aminopenicillin
- Aminopenicillin + ß-lactamase inhibitor
- Ureidopenicillin + ß-lactamase inhibitor
- Carboxypenicillin

- Cephalosporin 1st generation
- Cephalosporin 2nd generation
- Cephalosporin 3rd generation
- Cephalosporin 4th generation
- Carbapenem
- Metronidazole
- Fluoroquinolone
- others: ______________________

17. Is there a standard duration of the perioperative antibiotic prophylaxis?

- no
- single shot
- 24-48 hours
- 3-5 days
- 6-10 days
- >10 days

18. If not, which factors influence the duration of antibiotic prophylaxis? (several answers can be chosen)

- Antibiotic pre-treatment
- Pre-existing conditions, e. g. cachexia, ascites
- Known colonization of multi-resistant bacteria
- Age
- Intra-abdominal patch
- Course of C-reactive protein
- Course of procalcitonin
- Donor-specific risk factors, e.g., length of hospital stay
- Ascites after transplantation
- Presence of abdominal patch
- Presence of central venous catheter
- split organ
- other: ______________________

19. Is there a standard duration of the antibiotic therapy after considering the risk factors?

- no
- single shot
- 24-48 hours
- 3-5 days
- 6-10 days
- >10 days

20. Which is your standard escalation antibiotic therapy in case of suspected infection under prophylaxis (several answers can be chosen)?

- Carbapenem
- Ureidopenicillin + ß-lactamase inhibitor
- Cephalosporine 3rd generation
- Cephalosporine 4th generation

- Aminoglycoside
- Fluoroquinolone
- Vancomycin
- Linezolid
- Colistin
- Metronidazole
- other: ______________________

21. Do you use a standard anti-fungal peri-operative prophylaxis?

- no
- Fluconazole
- Posaconazole
- Voriconazole
- Liposomal amphotericin b
- Caspofungin
- other: ______________________

22. If you do not use an anti-fungal prophylaxis, are there risk factors that lead to anti-fungal prophylaxis on an individual basis? (several answers can be chosen)

- Antibiotic pre-treatment
- Pre-existing conditions
- Positive fungal cultures (colonisation)
- Donor-specific risk factors, e.g., length of hospital stay
- Age
- Intra-abdominal patch
- Re-operation
- Massive blood transfusion
- Course of C-reactive Protein
- Course of procalcitonin
- other: ______________________

23. If you use an anti-fungal prophylaxis, how long do you administer it?

- we do not use an anti-fungal prophylaxis
- 24-48 hours
- 3-7 days
- 14-28 days
- >28 days

24. Which are preferred biomarker to monitor bacterial infection? (several answers can be chosen).

- C-reactive Protein
- Procalcitonin
- Interleukin 6
- Interleukin 8
- Leukocyte count
- other: ______________________

25. Do you isolate your patient after the transplantation?

- no
- yes, period of time: ______________________

26. Do you use standardized surveillance strategies? (several answers can be chosen)

- no
- ascites
- tracheal secrete
- blood
- urine
- skin swabs
- rectal swabs
- preservation fluid of the graft
- nose swabs
- throat swabs
- other: ______________________

27. What is your standard antiviral regimen?

- Aciclovir prophylaxis
- Ganciclovir prophylaxis
- Pre-emptive therapy in case of viraemia
- We do not use a standard antiviral regimen.
- other: ______________________

28. The standard CMV prophylaxis is for:

- all patients
- both CMV positive
- recipient positive
- donor positive
- both negative

29. Do you want to add something? ______________________

30. If you want, you can name your hospital here. ______________________
